# Supplementary figures and images for: Evolutionary rescue of spherical mreB deletion mutants of the rod-shape bacterium Pseudomonas fluorescens SBW25
Source: eLife. 2025 Mar 31;13:RP98218. doi: 10.7554/eLife.98218 (PMC11957537; doi:10.7554/eLife.98218)

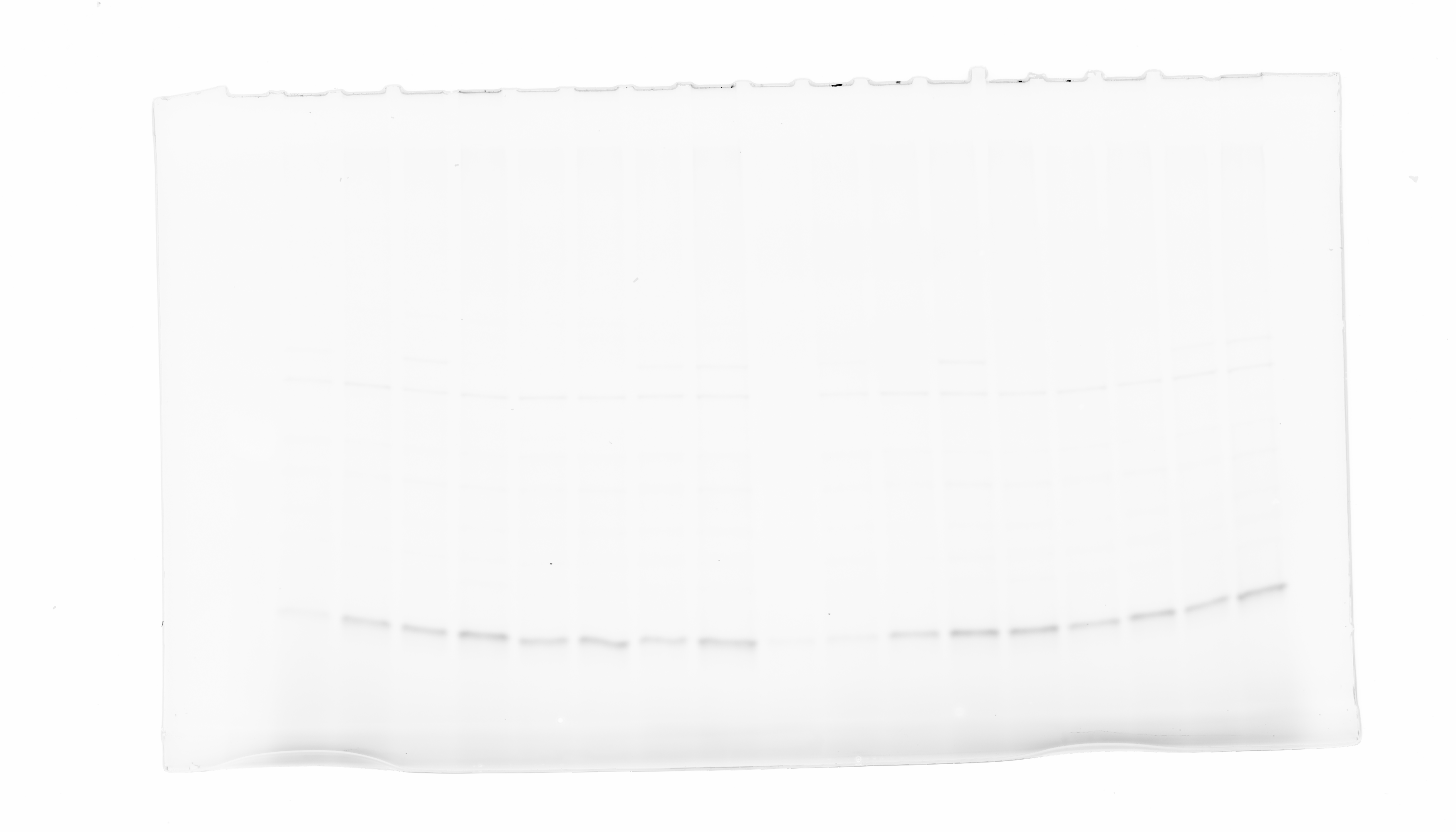

Supplement: Figure 4—source data 1. [file elife-98218-fig4-data1.zip › Figure 4 - source data 1 98218R2.tif]

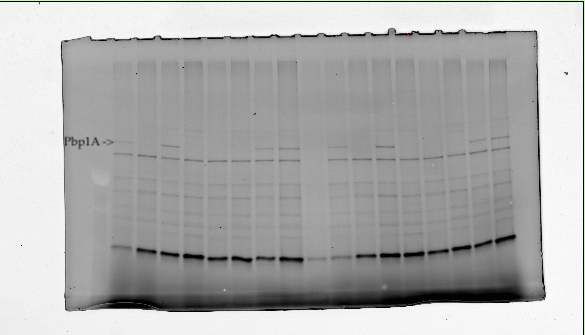

Supplement: Figure 4—source data 2. [file elife-98218-fig4-data2.zip › Figure 4 - source data 2 98218.tiff]
