# Supplementary material for: Evolutionary rescue of spherical mreB deletion mutants of the rod-shape bacterium Pseudomonas fluorescens SBW25
Source: eLife. 2025 Mar 31;13:RP98218. doi: 10.7554/eLife.98218 (PMC11957537; doi:10.7554/eLife.98218)
Supplement: Supplementary file 1. [file elife-98218-supp1.docx]

**Supplementary file 1**. Mutations identified in derived lines at generations 500 and 1,000.

|  | Mutation | | | |
| --- | --- | --- | --- | --- |
|  | Gene | Mutation | Freq @ 500 | Freq @ 1,000 |
| Line 1 | *pbp1A* | D484N (GAT->AAT) | 74% (n = 118)^1^ | 78% (n = 129) |
| Line 2 | *ftsA* | H346R (CAC->CGC) | 71% (n = 105) | 72% (n = 114) |
|  | *ctpA* | T174P (ACC->CCC) | 66% (n = 93) | 60% (n = 106) |
| Line 3 | *pbp1A* | D721A (GAC->GCC) | 99% (n = 75) | 87% (n = 78) |
| Line 4 | *pbp1A* | G719D (GGT->GAT) | 60% (n = 90) | ND^2^ |
|  | *pbp1A* | T362P (ACC->CCC) | ND | 98% (n = 100) |
| Line 5 | *ftsZ* | D176A (GAC->GCC) | 48% (n = 147) | ND |
| Line 6 | *pbp1A* | N698K (AAC->AAG) | 100% (n = 103) | 100% (n = 52) |
| Line 7 | deletion | 5399934-5403214 | 30% (n = 34) | 96% (n = 112) |
|  | *ftsE* | T188A (ACC->GCC) | ND | 63% (n = 125) |
| Line 8 | *pbp1A* | del A at 450,515 | 39% (n = 94) | ND |
|  | *ctpA* | T174P (ACC->CCC) | ND | 73% (n = 105) |
| Line 9 | *pbp1A* | G580D (GGC->GAC) | ND | 42% (n = 86) |
|  | *pbp1A* | W322* (TGG->TGA) | ND | 40% (n = 111) |
| Line 10 | *pbp1A* | T362P (ACC->CCC) | 32% (n = 111) | ND |
|  | *pbp1A* | G580D (GGC->GAC) | ND | 36% (n = 100) |

^1^Percentage of reads, n = number of reads.

^2^ND: no mutation detected.
