# Supplementary material for: Evolutionary rescue of spherical mreB deletion mutants of the rod-shape bacterium Pseudomonas fluorescens SBW25
Source: eLife. 2025 Mar 31;13:RP98218. doi: 10.7554/eLife.98218 (PMC11957537; doi:10.7554/eLife.98218)
Supplement: Supplementary file 2. [file elife-98218-supp2.docx]

**Supplementary file 2**. Mutations in *pbp1A* at generation 50.

| Line | Colonies with mutation  (of 16 colonies) | Nucleotide position  in PBP1a | Nucleotide change | Genomic Position | Protein-level change |
| --- | --- | --- | --- | --- | --- |
| 1 | 16 | 1450 | G>A | 449754 | D484N |
| 2 | 1 | 2114 | T>C | 449090 | F705S |
| 2 | 1 | 1814 - 1815 | Insertion | 449389 - 449390 | fs 605 |
| 3 | 3 | 1814 - 1815 | Insertion | 449389 - 449390 | fs 605 |
| 4 | 2 | 1148 - 1149 | Insertion | 450055 - 450056 | fs 383 |
| 4 | 1 | 1818 - 1819 | Deletion | 449385 - 449386 | fs 606 |
| 6 | 4 | 2032 | A>C | 449172 | T678P |
| 6 | 1 | 1084 | A>C | 450120 | T362P |
| 7 | 1 | 922 - 923 | Insertion | 450281 - 450282 | L308P |
| 8 | 2 | 689 | Deletion | 450515 | fs 230 |
| 8 | 1 | 1339 | A>C | 449865 | S447R |
| 9 | 2 | 996 | A>G | 450208 | W>STOP |
| 10 | 1 | 1739 | G>A | 449465 | G580D |
